# Segmentation window of speech information processing in the human auditory cortex

**DOI:** 10.1038/s41598-024-76137-y

**Published:** 2024-10-24

**Authors:** Minoru Hayashi, Tetsuo Kida, Koji Inui

**Affiliations:** 1 Department of Interdisciplinary Science and Engineering, School of Science and Engineering, Meisei University, Tokyo, 191-8506 Japan; 2 Department of Functioning and Disability, Institute for Developmental Research, Aichi Developmental Disability Center, Kasugai, Japan; 3 Section of Brain Function Information, National Institute for Physiological Sciences, Okazaki, Japan

**Keywords:** Continuous speech, Speech perception, Auditory evoked magnetic fields, Temporal segmentation window, Superior temporal area, Neuroscience, Psychology

## Abstract

Humans perceive continuous speech signals as discrete sequences. To clarify the temporal segmentation window of speech information processing in the human auditory cortex, the relationship between speech perception and cortical responses was investigated using auditory evoked magnetic fields (AEFs). AEFs were measured while participants heard synthetic Japanese words /atataka/. There were eight types of /atataka/ with different speech rates. The durations of the words ranged from 75 to 600 ms. The results revealed a clear correlation between the AEFs and syllables. Specifically, when the durations of the words were between 375 and 600 ms, the evoked responses exhibited four clear responses from the superior temporal area, M100, that corresponded not only to the onset of speech but also to each group of consonant/vowel syllable units. The number of evoked M100 responses was correlated to the duration of the stimulus as well as the number of perceived syllables. The approximate range of the temporal segmentation window limit of speech perception was considered to be between 75 and 94 ms. This finding may contribute to optimizing the temporal performance of high-speed synthesized speech generation systems.

## Introduction

Humans perceive continuous speech signals as discrete sequences. To understand the mechanism of speech perception in the human brain, it is necessary to clarify the segmentation window of speech processing, which is the temporal gateway for speech information processing. These sequences have millisecond-order dynamics and electroencephalograms (EEGs) and magnetoencephalograms (MEGs) with a high temporal resolution^1–6^ are suitable to investigate speech perception mechanisms non-invasively. MEGs have an advantage of high spatial resolution for localizing brain activities and hold potential for analyzing speech dynamics, such as speech processing units^7–12^ and the temporal segmentation of speech information processing.

Previous studies using birdsong and speech have suggested that a unit of song is a vocalization burst of 10–1000 ms^13–18^. In the auditory system, the latency of N30 event-related potential (ERP) components in monkeys is considered to be approximately three-fifths of the corresponding human ERP component N48^19^. Recent research has shown that the latency of the auditory N1 component is approximately 100 ms for humans, and 50 ms for rhesus monkeys^20^. Longer auditory cortical processing times favor the analysis of time-varying acoustic stimuli such as speech perception.

In the field of neuroscience, the exploration of understanding speech processing units in the human brain through non-invasive means continues unabated^11,12,21–26^. Studies using neural oscillation suggest that the time span of speech processing units is approximately 25–250 ms^27–34^. Speech processing operates on multiple hierarchical levels, necessitating the coexistence of various higher-order processing windows, all of which are important^35–37^. For example, a long-time window for attention processing has been proposed^38^. However, when simply listening to continuously changing sounds, such as words, the temporal segmentation window of speech information processing for speech perception especially in relation to auditory evoked magnetic fields (AEFs), remains unclear.

Previous studies on AEFs have used several phonemes, consonants, vowels, syllables, and amplitude-modulated noises, as well as spectral transitions of one and two syllables^11,12,39–43^. According to these studies, AEFs are different for each phoneme, consonant, vowel, and syllable. The effect of the differences in consonant duration, sound pressure, vowel amplitude, each syllable component, and left-right hemisphere differences should also be considered. Thus, to solve the speech perceptual temporal segmentation window problem, synthetic speech is required to precisely control the acoustic physical structure of speech sounds. In this study, we investigated the relationship between the AEFs and subjective auditory perception using synthetic speech. As auditory M100, also known as N100, can be sensitive to subtle changes in sound features^44^ including a silent gap^45^, we investigated effects of variations in duration of speech on evoked M100.

The experiment for this study was designed with three basic concepts. First, it aimed to precisely control the acoustic physical structure, particularly the temporal structure, of the speech stimuli used in the experiment. Second, the auditory cortex response was measured for various speech rates of the stimuli to clarify the temporal structure of the speech processing limit. Third, our study aimed to determine how accurately participants could hear and perceive the speed of the speech stimuli, to clarify its correlation with the auditory cortex response. Using these basic concepts, the range of the temporal segmentation window in the perception of continuous speech word was investigated.

## Results

**Fig. 1:**
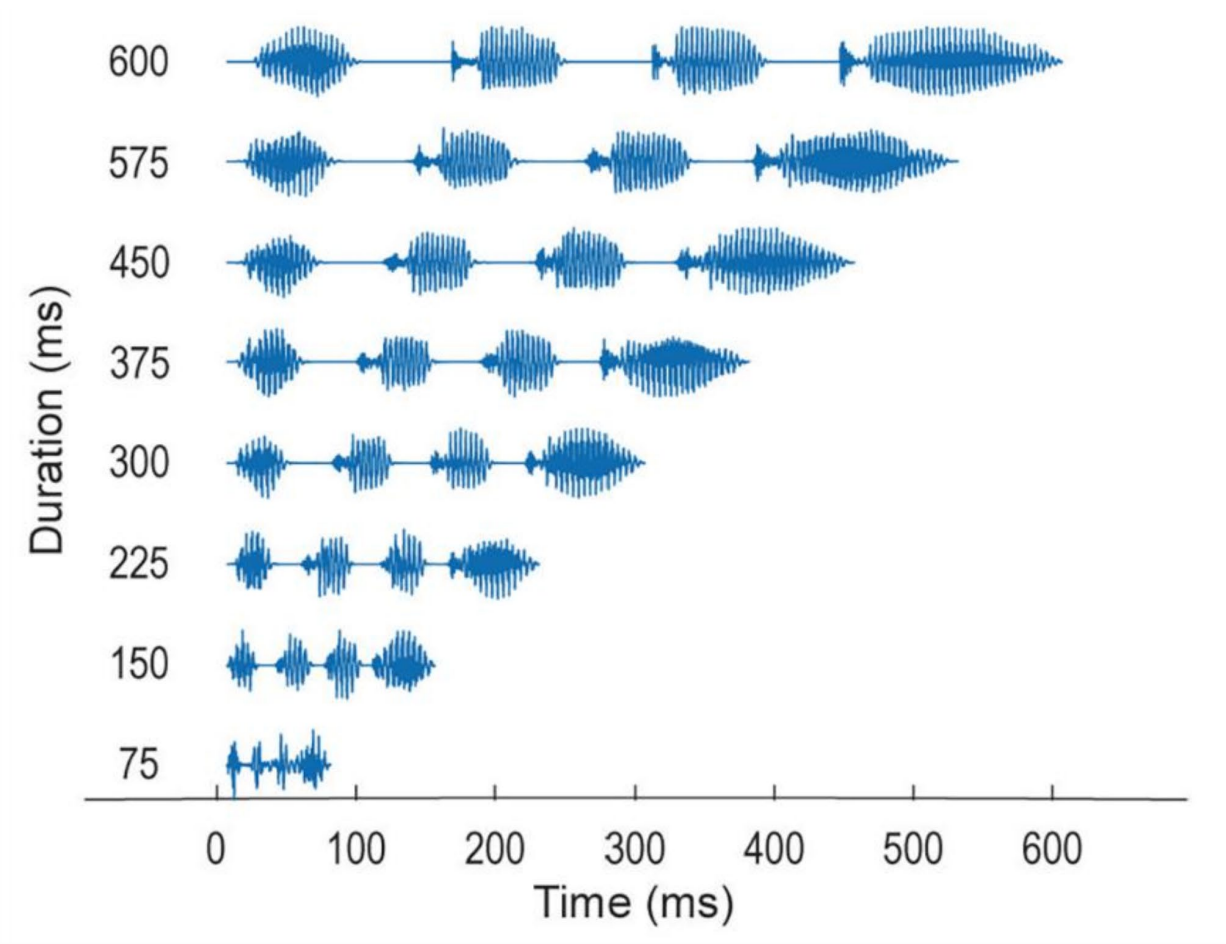
Eight speech stimuli with different speech rates. Waveforms are presented in milliseconds.

To identify the segmentation window range more precisely, a time-stretching method was employed to increase the speech rate while preserving the phonological speech structure. We generated eight types of speech stimuli with different speeds. Figure 1 illustrates the waveforms of the eight speech stimuli, with different speech rates in milliseconds. These eight types of speech stimuli that consisted of seven phonemes and four syllables with different durations from 75 to 600 ms were randomly presented to participants and the AEFs were recorded.

**Fig. 2:**
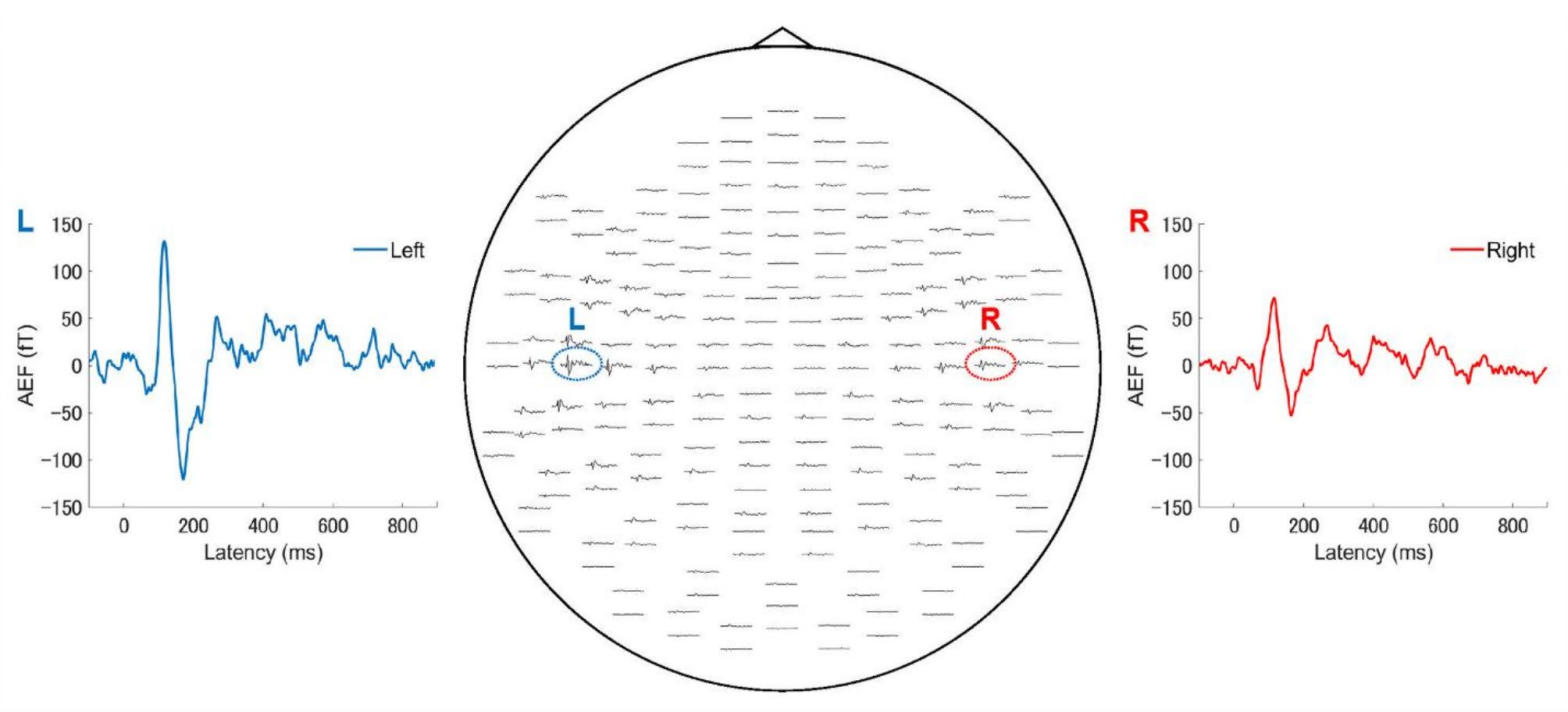
Example of auditory evoked magnetic fields following the 600-ms stimulus. Data obtained from a single participant. The left and right panels display the enlarged waveforms of the channel with the largest response from both hemispheres. The middle panel illustrates the magnetic waveforms of all 204 channels. AEF, auditory evoked magnetic field.

**Fig. 3:**
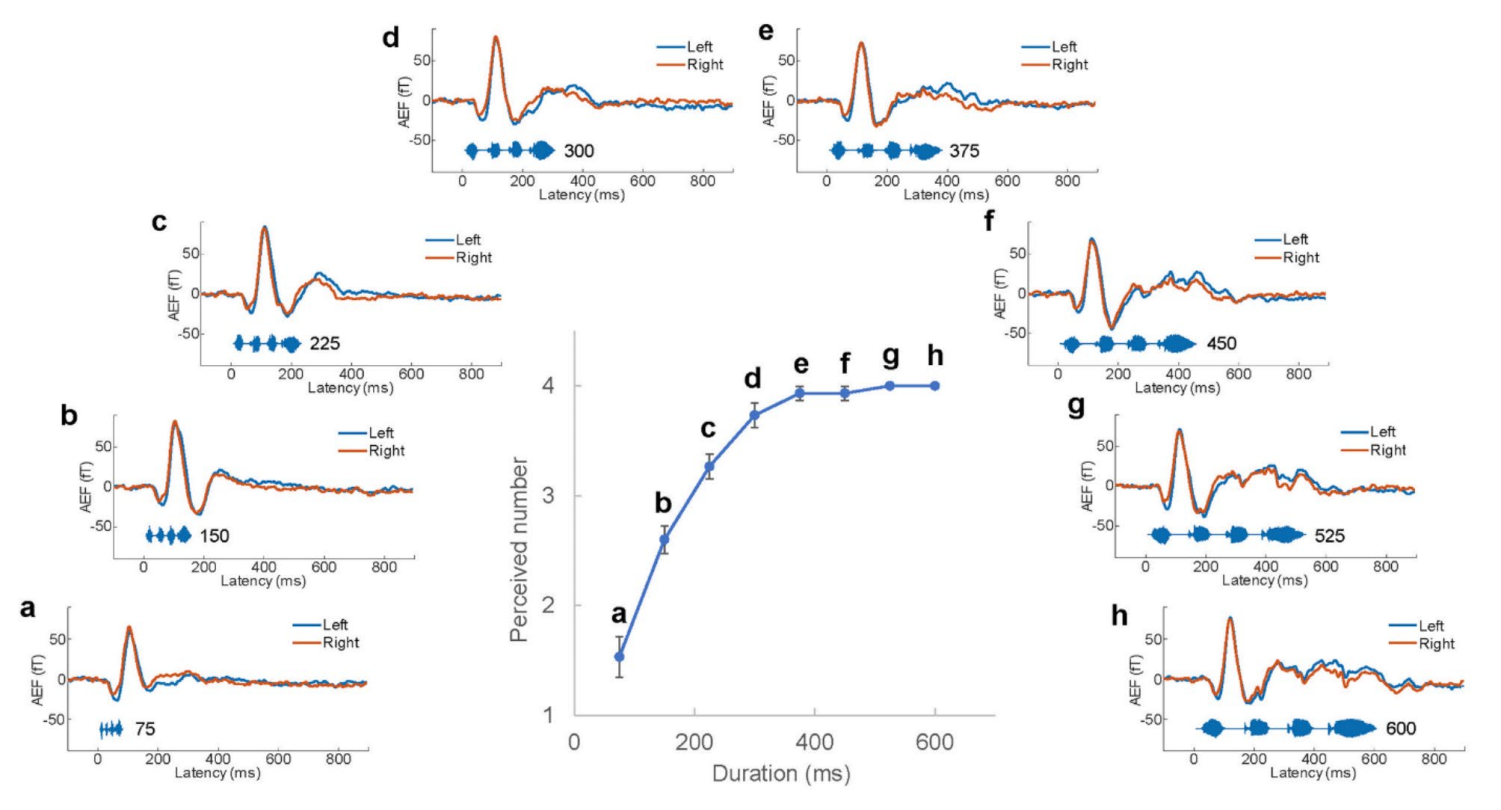
Relationship between the perceived number and duration of speech stimuli. The middle graph illustrates the relationship between the perceived number of stimuli and the stimuli duration. The y-axis in the graph indicates the perceived number, and the x-axis indicates duration. The error bars represent ± 1 standard error of the mean across all participants. The grand-averaged AEF waveforms for each stimulus are shown in panels (**a**–**h**). AEF, auditory evoked magnetic field.

**Fig. 4:**
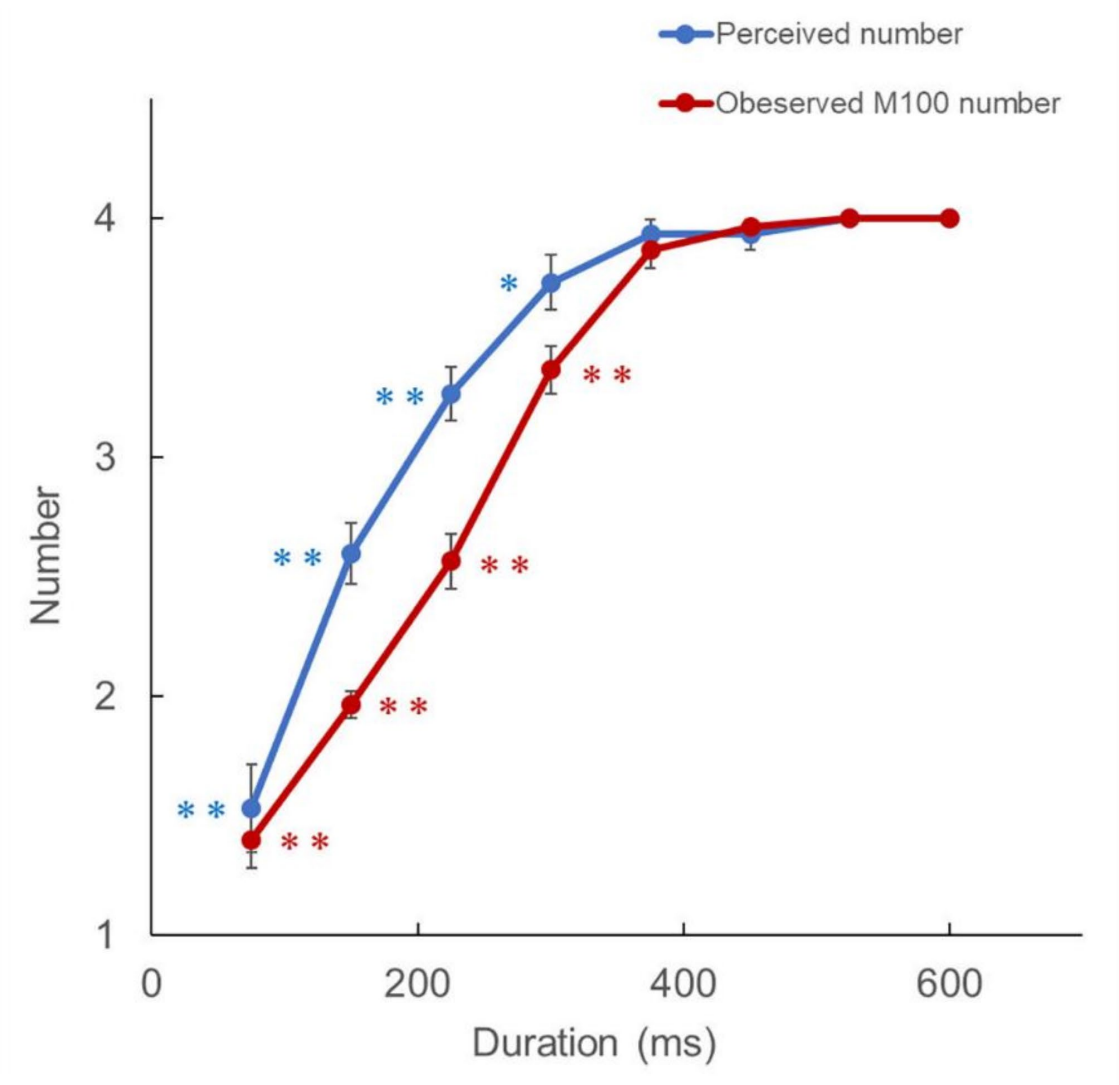
Correlation between perceived number and observed M100 number. The y-axis of the graph indicates both the perceived number and the observed M100 number, and the x-axis represents duration. The error bars denote ± 1 standard error of the mean across all participants. The relationship was assessed using Wilcoxon signed-rank tests with asymptotic significance probability for the 600-ms stimulus. The asterisks indicate the significance of the results. *, *p* < 0.05; **, *p* < 0.01.

An example of the AEFs elicited by the 600 ms speech stimulus is presented in Fig. 2. Grand-averaged waveforms of the AEFs are shown in Fig. 3 **a**–**h**. The middle panel in Fig. 3 shows the number of perceived sound chunks plotted against the stimulus duration. There was a significant positive correlation between the number of perceived stimuli and the duration of the stimuli (*r* = 0.82, *P* = 1.42 × 10^− 30^). As displayed in the AEF waveforms in Fig. 3, there was a clear response approximately 100 ms after stimulus onset, defined as M100_1_. Following M100_1_, smaller responses were observed in the same direction. As the stimulus duration increased M100_1_, M100_2,_ M100_3,_ and M100_4_ all became clearer. When the significance of the M100 component was defined as those with amplitude greater than 4 standard deviations (SDs) from baseline, the number of M100 components in the AEF waveforms increased with an increase in stimulus duration. Figure 4 illustrates the strong correlation between the duration of stimuli and observed M100 number (*r* = 0.91, *P* = 9.29 × 10^− 47^). The perceived number and observed M100 number strongly correlated (*r* = 0.86, *P* = 2.18 × 10^− 36^).

**Fig. 5:**
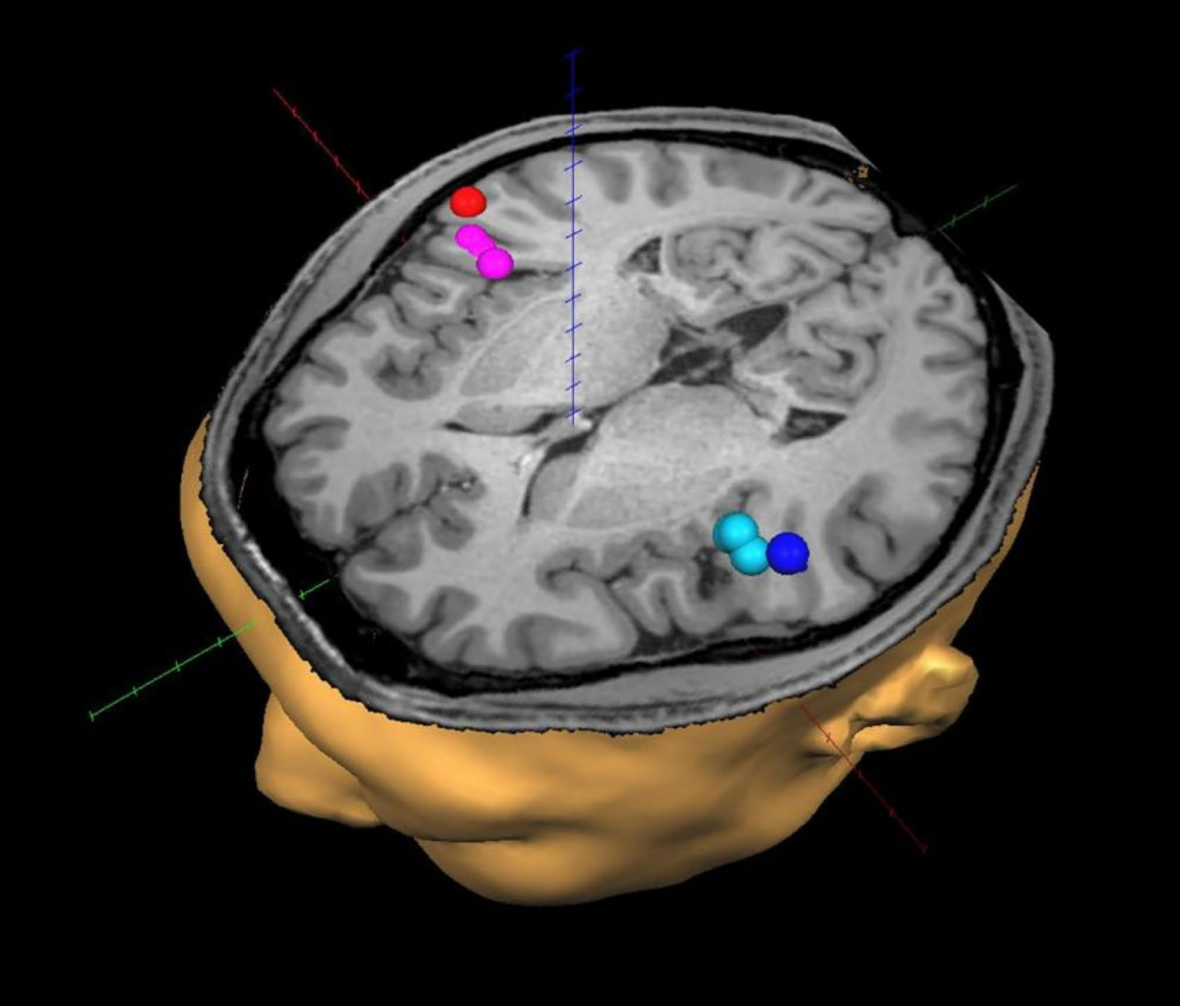
Dipole locations of the main auditory evoked magnetic fields components. The estimated source location of M100 in a representative participant. The dipole locations for the first syllable are shown in blue (left hemisphere) and red (right hemisphere), and those for the second to fourth syllables are shown in cyan and magenta.

**Fig. 6:**
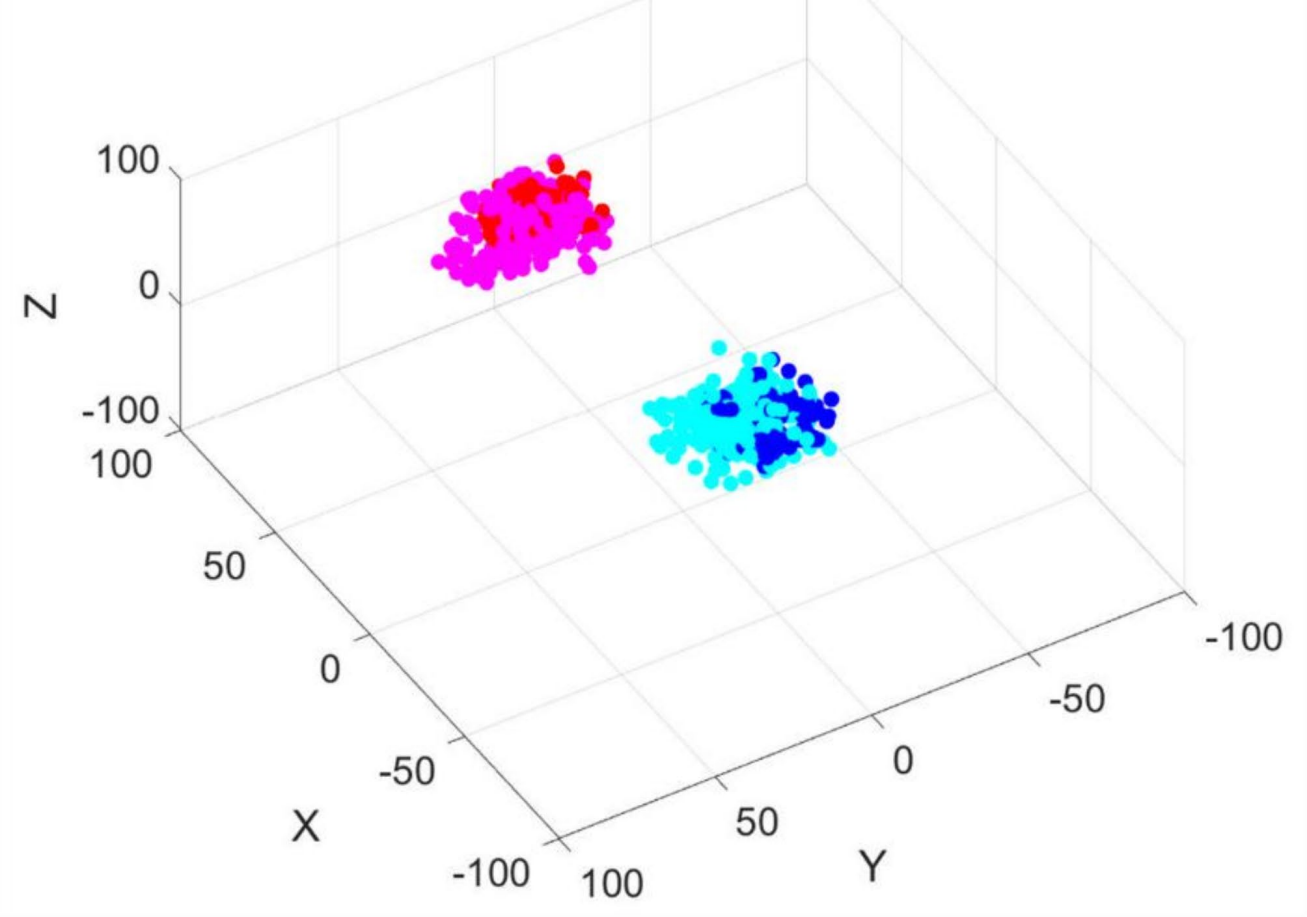
Estimated location of the M100 component for the first syllable and subsequent syllables. The dipole locations for all stimuli in all participants are superimposed. In the left hemisphere, the blue dots indicate the estimated location of the M100 dipole for the first syllable, and the cyan dots indicate those for the second to fourth syllables. In the right hemisphere, the red dots indicate the location of the first syllable, whereas the magenta dots indicate those of the second to fourth syllables.

Figure 5 displays the dipole locations of the main AEF component, M100, for a single participant. Figure 6 illustrates the location of estimated dipoles for all participants. As shown in Figs. 5 and 6, the dipole in response to the first syllable was located more lateral to those of subsequent syllables. The results of the discriminant analysis indicated the difference was significant (Table 1).

**Table 1:** Differences in dipole location between the first syllable and subsequent syllables.

	Left hemisphere		Right hemisphere	
	Wilks’ Lambda	*P* value	Wilks’ Lambda	*P* value
1 vs. 2	0.91	1.3 × 10^− 4^	0.95	4.6 × 10^− 3^
1 vs. 3	0.76	2.1 × 10^− 13^	0.96	2.2 × 10^− 2^
1 vs. 4	0.81	1.5 × 10^− 10^	0.87	5.1 × 10^− 7^
2 vs. 3	0.97	0.07	0.10	0.82
2 vs. 4	0.98	0.18	0.98	0.15
3 vs. 4	0.99	0.51	0.97	0.06

The results of the discriminant analysis are listed.

The *P* values are not corrected for multiple comparisons.

## Discussion

The relationship between temporal information, perceived number, speech rate, and the M100 component shown in Figs. 3 and 4 suggests that as the speech rate approached the normal speech rate, the number of perceived sound chunks approached four, with a significant M100 component for each syllable. The strong correlation of M100 with the perception of a sound chunk suggests that M100 reflects the audibility of sound chunks.

**Fig. 7:**
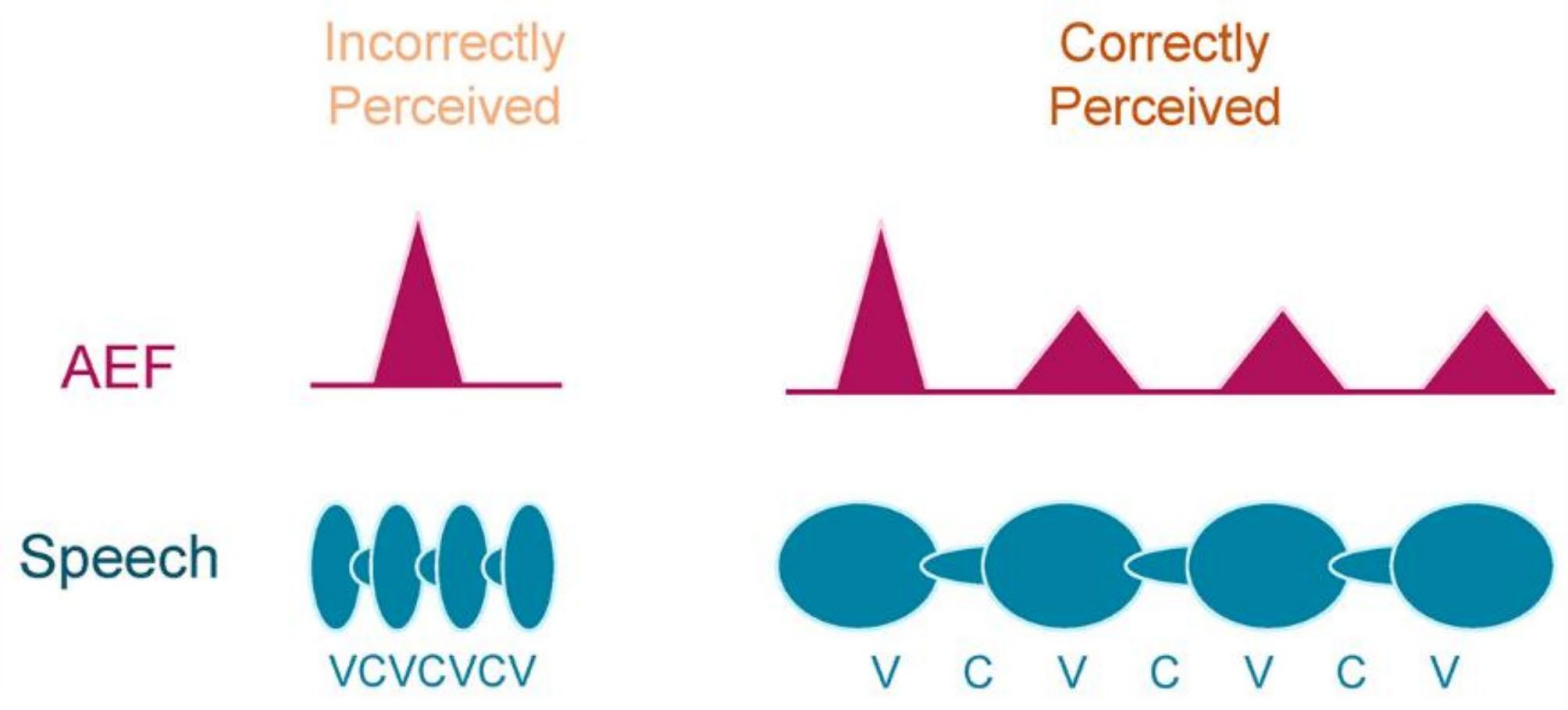
Model of the unit of speech perception. The upper and lower panels schematically show the neuromagnetic field response (auditory evoked magnetic field [AEF]) corresponding to speech waveforms composed of consonants (C) and vowels (V).

Regarding the perceived number of sound chunks, the participants could perceive all four syllables correctly for sound durations of 600 and 575 ms and almost correctly for 450 and 375 ms; however, from 300 ms, the audible number decreased as the duration shortened. The boundary of perception was between 375 and 300 ms. The similar boundary for the occurrence of all the four M100 was between 375 and 300 ms (Fig. 4). The speech stimulus /atataka/ used in this experiment has four syllables. As the average syllable lengths for the 375- and 300-ms sounds were 93.75 ms and 75.00 ms respectively, these results indicated the range of the temporal segmentation window for speech perception was approximately 75–94 ms. The strong correlation between M100 and sound perception suggests that the AEF was discretely observed for continuous acoustic physical quantities. The results also indicated that speech processing of consonants and vowels occurred simultaneously when hearing a single syllable unit. This suggests that syllables are functional units of speech perception when humans hear continuous acoustic signals in the form of discrete sequences. Figure 7 illustrates the relationship between the unit of speech perception and AEFs.

The estimated dipoles of M100_1_ were located in the superior temporal gyrus (Heschl’s gyrus)^41,46–50^ in both hemispheres. The significantly more medial location of the dipoles of M100_2_, M100_3,_ and M100_4_ compared to M100_1_ suggests that distinct neural groups contribute to speech information processing of the first and subsequent syllables, at least in part. One possible explanation is a novelty-related response to the first syllable, which originates in the lateral part of the Heschl’s gyrus^51^.

This experiment utilized synthetic speech stimuli that precisely controlled the type of consonant, vowel, duration, and other physical structure components of speech sound and successfully measured clear responses to each syllable unit. We believe this approach contributes to understanding the speech information processing function. Due to the recent advancements in synthesized speech quality, high-speed speech reproduction has become popular for pursuing timely performance^52–54^. Understanding the optimal speech rate and functional units of speech processing in the human auditory system is essential. The auditory system is temporal-dependent, and brain rhythms are crucial for processing auditory information^55^. In particular, slow synchronization is essential for speech perception, especially under conditions involving a high volume of acoustic information^56^.

The stimuli used in this experiment were constructed as VCVCVCV (C: consonant, V: vowel) sequences, consisting of seven phonemes (V, C, V, C, V, C, V) and four syllables (V, CV, CV, CV). The results indicated that the number of perceived auditory chunks corresponded to the number of syllables (V, CV, CV, CV), which is consistent with previous syllable perception studies. The time point reference for the stimuli used in this experiment was the point of consonant*/*vowel spectral transition, which has been shown to be crucial for syllable perception in a study by Furui^8^. Additionally, a psychophysical experiment by Ghitza and Greenberg^57^ on the effect of changes in syllable rhythm on sentence intelligibility indicated that the temporal range contributing to intelligibility was 40–120 ms. The time range observed in the present study, 75–94 ms, is in the center of this range, suggesting a relationship with syllable perception. Furthermore, the present results are consistent with previous studies investigating the relationship between the second syllable of disyllabic stimuli and the M100 response, which have shown that the spectral transition of the syllable corresponds to the M100 component in disyllabic stimuli^12^. The present results are also in line with earlier findings that the syllable corresponds to the M100 component in monosyllabic plosive consonant stimuli^11,58^.

It is well known that the human auditory system processes speech information at different hierarchical levels, and thus, there are multiple perceptual windows. In a study using intracranial EEG recordings, Norman-Haignere et al.^36^ demonstrated diverse timescales ranging from 50 to 400 ms with distinct functional properties: the short-integration window (< 200 ms) showed prominent spectro-temporal modulation selectivity, while the long-integration window (> 200 ms) showed prominent category selectivity. The limit of the segmentation window in the present study aligns well with their short window in both timescale and function. Teng et al.^35^ indicated a similar dual-scale model of speech processing based on behavioral experiments. Furthermore, Teng et al.^37^ used MEG to present non-speech modulated sounds, noting that the auditory system has two different time-scale processing modes. The sampling scales of the low-frequency theta band and the high-frequency gamma band may be separated by the mid-frequency alpha band. The segmentation window limit of speech perception obtained from the results of the present study may potentially help explain these different temporal processing mechanisms. In another study by Marinato and Baldauf^38^ on the parsing of speech signals in an attentional setting, they proposed that long processing windows are important for the successful recognition of speech repetition targets. It is likely that the M100-based analysis primarily reflects processing within earlier time windows in the primary and/or secondary auditory cortex. An MEG study by de Vries et al.^59^ showed that 8–14 Hz oscillations in the auditory cortex are important for top-down control of attention in the recognition of complex naturalistic soundscapes. The frequency of oscillations is roughly consistent with the time window of 75–94 ms obtained in the present study, and these results may therefore be due to a common mechanism. A previous study revealed significant relationships of neural oscillations with word, or syllable rhythms^60^. Therefore, future studies are necessary to find relationships between M100 and neural oscillations during speech perception, including the possibility that some oscillatory activities may be obtained without clear M100 responses. It also should be noted that there is a link between cortical activities and phonemes^61,62^. In this experiment, the duration of words was set to 600 ms, with syllables having durations of approximately 150 ms based on natural speech rates. This range of syllable duration includes phonemes spoken slowly and words spoken rapidly. It should be considered whether the range of 75–94 ms can account for the perceptual thresholds of words, syllables, and phonemes. Additionally, whether multivariate temporal response functions^63^ and spectral domain analyses could clarify AEFs more distinctly in cases involving diverse responses should be considered.

The preset study employed a single Japanese word composed of a VCVCVCV sequence structure. Thus, there are limitations when extending our findings to continuous speech in general. However, the current method of creating synthetic speech stimuli, which allows for precise control of various acoustic physical parameters including duration, can be applied to any language or any form of continuous speech. As detailed in the **Speech stimuli** of the **Methods**, our experiment utilized synthetic speech stimuli with precisely controlled duration and other temporal structures, types of consonants, vowels, fundamental frequency, and other acoustic physical structure of speech sounds. It is both fascinating and noteworthy to investigate cortical responses and auditory perception using synthetic speech stimuli. This straightforward yet highly precise approach provides clear and practical insights.

To uncover more extensive and universal functional processing units, future research would benefit from investigating units such as C, V, CVC, CVVC, CVCC, and CCVC, along with synthetic speech stimuli that mimic common acoustic physical structures of speech. Additionally, the approach and findings of this study are expected to elucidate the perceptual temporal window and more, thereby providing a valuable foundation for advancing our understanding of auditory speech perception.

## Conclusions

The human brain has temporal limitations in processing speech information. Hence, a temporal window is necessary for segmenting speech. The present study was conducted to identify the precise temporal range of the perceptual window that is most significant for human speech perception using MEG measurement and a subjective evaluation of speech perception. The results indicated that the temporal segmentation window limit was approximately 75–94 ms when humans hear continuous acoustic signals as discrete sequences. We also revealed a clear correlation between AEFs and syllables when participants listen to speech stimuli. This temporal window is the master gate of speech perception in humans. This finding not only serves as a foundation for understanding the mechanisms of speech perception but also holds the potential for contributing to optimizing the temporal performance of high-speed synthesized speech generation systems. Clarifying speech perception using synthetic speech with precisely controlled acoustic physical structure is expected to provide a breakthrough in understanding the mechanisms of human communication.

## Methods

### Ethics statement

The experimental protocol was designed in accordance with the Declaration of Helsinki, and was approved by the Ethics Committees of Meisei University, Tokyo, Japan, and the National Institute for Physiological Sciences, Okazaki, Japan. All the participants provided written informed consent prior to participating in the study.

### Participants

Fifteen healthy participants (mean age: 40.8 ± 12.7 years; 4 women, 11 men) with normal hearing ability were included. The mean score of the Edinburgh Handedness Inventory^64^ was 73.2 ± 52.7 (13 right-handed participants, mean score: 93.7 ± 7.5; 2 left-handed participants, mean score: −60.0). All participants were native Japanese speakers without any history of hearing impairment or neurological or psychiatric disorders, according to self-reports.

### Speech stimuli

From our previous study, it is evident that the M100 component dipoles vary with vowels^40^; thus, the speech stimulus used /a/ as vowels. Additionally, because the M100 elicited by syllables with a consonant (C) and vowel (V) at the beginning of a word vary with consonant duration and type, the responses to consonants and vowels overlap^11^. Hence, the beginning of words for this experiment had the /a/ vowel, with the plosive consonants /t/ and /k/ chosen for mid-word positions. Therefore, the experiment employed the seven phonemes and the four-syllable Japanese word /atataka/, meaning “warm,” as the speech stimulus. The speech synthesis engine “Cralinet” (NTT, Tokyo, Japan) synthesized the stimuli, employing the time-stretch method to generate stimuli with eight speech rates while maintaining consistent average sound pressure.

The sound pressure level of the stimulus within the earpiece fitted in the participant’s ear was measured using a precision sound level meter (NL-32, RION, Tokyo, Japan) with a custom-order precision coupler to fit the earpieces. The sound pressure level of the background noise was measured using the precision sound level meter (NL-32, RION). The delay in time from the stimulus presentation to the time at which the stimuli reached the earpieces was corrected as follows: The electrical and electronic system delays were approximately 3 ms, confirmed via an oscilloscope. The ear-tube length was approximately 138 mm with a delay time of approximately 4 ms, which was calculated from the approximation formula of sound transmission speed *y* = 331.5 + 0.6*x*, where *x* and *y* indicate the temperature (°C) and sound speed (m/s), respectively. The total delay of the sound stimulation system was 7 ms. Figure 1 illustrates the eight speech stimulus waveforms with different speech rates. The standard speech reference time was set from the spectral transition represented by the dynamic measure ($\:D$), obtained by calculating the sum of the squares of each cepstrum coefficient (*C*_*i*_). A dynamic measure was utilized as the time reference point for each phoneme boundary of continuous speech (Eq. 1).

1 $$\:D\left(t\right)\:=\:\:\sum\:_{i\:=\:1}^{N}{\left\{{C}_{i}\left(t\right)\right\}}^{2}\:$$

During MEG measurements, speech stimuli were presented to participants seated inside a magnetically shielded room, and AEF signals were recorded on the acquisition computer. Stimuli were presented using earpieces (Model TIP-300; Nicolet Biomedical, Madison, WI, USA), finely adjusted so they could be heard in the midline with both ears and then presented binaurally. The delivery timing of each stimulus was controlled using personal computers and software. Sound pressure was regulated using an audiometer (AA-71; RION). The stimulus conditions were as follows: averaging number: 100; presentation order: random; inter-stimulus intervals: 1500–2500 ms random; average sound pressure level: 65 dBA; and background noise level: 40 dBA.

### Experimental procedures

As a preliminary step before recording, five head-position indicator (HPI) coils were strategically placed on the participant’s head to obtain its precise location. The positions of the HPI coils, three anatomical landmarks (left preauricular, right preauricular, and nasion), and at least 30 points on the scalp surface were measured using a three-dimensional digitizer calibrated to permit comparison with magnetic resonance imaging (MRI) data. The experiments were performed in a quiet, magnetically shielded room. To minimize the impact of higher-order processing not directly related to auditory information processing, participants were instructed to watch a silent movie projected on a screen 1.5 m in front of them and ignore the sound throughout the MEG experiment. The silent movie was projected onto the screen using a projector (Mirage S+3K, Model 38-DSP102-74, Christie Digital Systems Inc., Kitchener, Canada) located outside the shielded room. Eight speech stimuli were randomly ordered, and at least 100 artifact-free epochs were averaged for each condition.

### Behavioral experiment

A subjective evaluation experiment was conducted prior to MEG recording. In this evaluation experiment, the measurement room, stimulus, and stimulus system were set up under a similar environment as that of the MEG experiment. Participants were instructed to indicate the number of audible sounds from the presented stimulus, determine what the stimulus sounded like, and write the perceived speech stimulus in letters. The eight stimuli with different durations were initially presented individually in the fast order and then individually in the reverse order.

### MEG measurement

The experiments were conducted in a magnetically shielded room. Brain magnetic fields were recorded using a 306-channel whole-head MEG system (Vector-view, ELEKTA Neuromag, Helsinki, Finland), comprising 102 identical triple sensor elements. Each sensor element was composed of two orthogonal planar gradiometers and one magnetometer coupled with a multi-superconducting quantum interference device, providing three independent measurements of magnetic fields. This study analyzed the MEG signals recorded from sufficiently powerful 204 planar-type gradiometers to detect the largest signal just over local cerebral sources^65–67^. Signals were recorded using a bandpass filter (0.1–330 Hz) and digitized at a sampling rate of 1000 Hz. Epochs with MEG signals larger than 3000 fT/cm were excluded from averaging. Analyses were conducted from 100 ms before to 900 ms after stimulus onset.

### MEG data analysis

For analyzing the most significant temporal information, raw MEG data were selectively averaged for each condition, and digitally filtered using a bandpass filter of 1.0–75 Hz with a notch filter of 60 Hz. The pre-stimulus period (−100 to 0 ms) was used as the DC baseline. The M100 component following the second to fourth syllable was evaluated with reference to the M100_1_ latency, ± 20 ms of the expected peak latency. The M100 component was defined as significant when the difference between the peak M100 amplitude and that of the preceding polarity-reversed component, M50, was greater than 4 SD of the pre-stimulus baseline. To analyze the spatial information of neural activity, dipole analyses of responses were performed using the Brain Electrical Source Analysis software package (BESA Research 5.3.7, GmbH, Grafefling, Germany) for computing theoretical source generators in a three-layer spherical head model. This method allowed the spatiotemporal modeling of multiple simultaneous sources over defined time intervals. The locations and orientations of the dipoles were calculated using an iterative least-squares fit. The location of the source of neural activity was estimated using a 40 ms range of 20 ms before and 20 ms after the maximum amplitude latency of the response to the first syllable. According to the time of maximum amplitude latency for the first note using acoustic physics, the time to the onset of the second, third, and fourth syllables (the CV syllable spectral transition time) was estimated using a 40 ms range, with 20 ms before and after.

### MRI

A series of structural images were also acquired from the participants on another day using a 3-T MRI scanner (Magnetom Verio; Siemens Ltd., Erlangen, Germany)^68^. The neuroimaging software Brain Voyager QX (Brain Innovation BV, Maastricht Netherlands) was used to align and superimpose the estimated dipole locations of neural activity from the MEG data on the MRI images.

### Statistical analyses

Spearman’s rho correlation coefficients were calculated with a significance probability test to evaluate the relationship between the number of perceived stimuli and the duration of the stimuli, the relationship between the duration of the stimuli and observed number of M100 components, and the relationship between the perceived number and observed number of significant M100 components. The Wilcoxon signed-rank test was used to evaluate the asymptotic significance of each stimulus for the 600-ms. A discriminant analysis was used to identify differences in the dipole location and orientation using the x-, y-, and z-coordinate positions and vector directions as variables. Statistical analyses were performed using SPSS (version 28.0; IBM Corp., Armonk, NY, USA).

## Data Availability

The data measured and analyzed in this study are available from the corresponding author upon reasonable request.
